# Supplementary material for: Quantum sensing of microRNAs with nitrogen-vacancy centers in diamond
Source: Commun Chem. 2024 May 6;7:101. doi: 10.1038/s42004-024-01182-7 (PMC11074114; doi:10.1038/s42004-024-01182-7)
Supplement: Supplementary file 2 — Supplementary Information [file 42004_2024_1182_MOESM2_ESM.pdf]

Supplementary Information for:

"Quantum sensing of microRNAs with nitrogen-vacancy centers in diamond"

Justas Zalieckas*[1], Martin M. Greve[1], Luca Bellucci[2,3], Giuseppe Sacco[4], Verner Håkonsen[5], Valentina Tozzini[2,3,6] and Riccardo Nifosì*[2,3]

[1]Department of Physics and Technology, University of Bergen, Allegaten 55, Bergen 5007, Norway.
[2]Istituto Nanoscienze - CNR, Piazza San Silvestro 12, Pisa 56127, Italy.
[3]Lab NEST Scuola Normale Superiore, Piazza San Silvestro 12, Pisa 56127, Italy.
[4]Scuola Internazionale Superiore di Studi Avanzati (SISSA), Via Bonomea 265, Trieste I-34136, Italy.
[5]NTNU NanoLab, Norwegian University of Science and Technology, Trondheim 7491, Norway
[6]Istituto Nazionale di Fisica Nucleare (INFN), sezione Pisa, Largo Pontecorvo 3, Pisa 56127, Italy

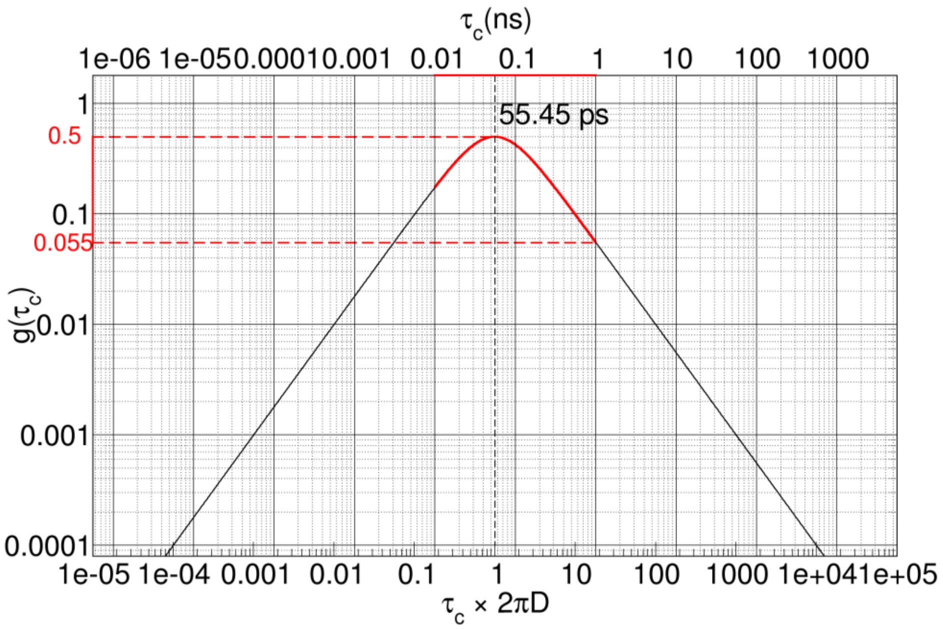

FIGURE S1. Plot of $g(\tau_c) = \frac{\omega_0 \tau_c}{(1+\omega_0^2 \tau_c^2)}$, where $\omega_0 = 2\pi D = 18.03$ GHz. The maximum of $g(\tau_c)$ takes on a value of ½ and occurs at $\tau_c = \frac{1}{\omega_0} = 55.45$ ps. The segments marked in red correspond to plausible values of τ_c and of $g(\tau_c)$.

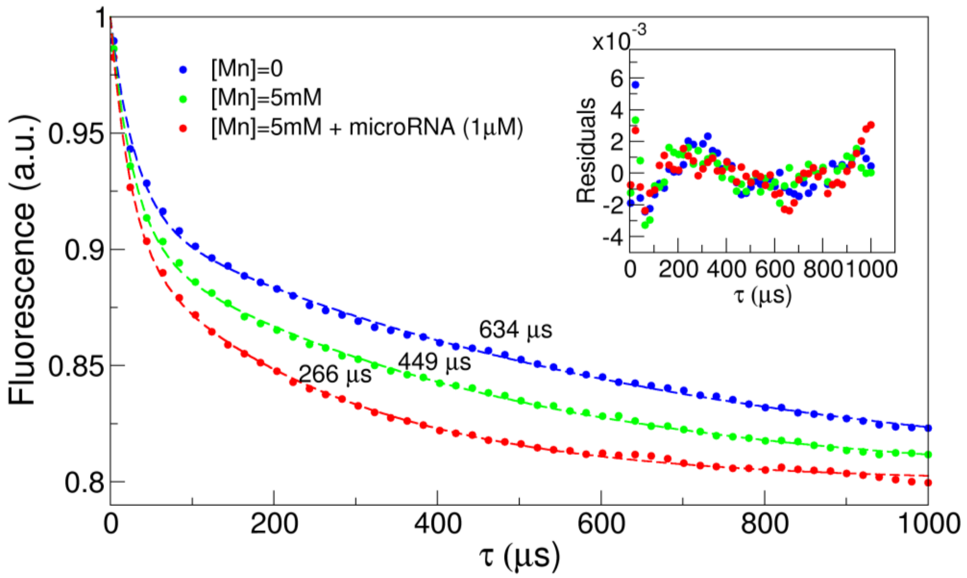

FIGURE S2. Measurement of the NVs relaxation time in water (blue dots), in the presence of Mn^{2+} at 5 mM (green dots), and with the addition of miR-21 at 1μM. The dashed curves represent fits using a bi-exponential function $Ae^{-\tau/T_{1,long}} + Be^{-\tau/T_{1,short}} + C$. Residuals are shown in the inset. The reported values are for the $T_{1,long}$, and correspond to $\Gamma_1 = 1.5, 2.2, 3.7$ GHz. The other fit parameters are, respectively: $T_{1,short} = 30.5, 28.2, 24.4$ μs and $A{:}B = 57{:}43, 52{:}48, 51{:}49$.

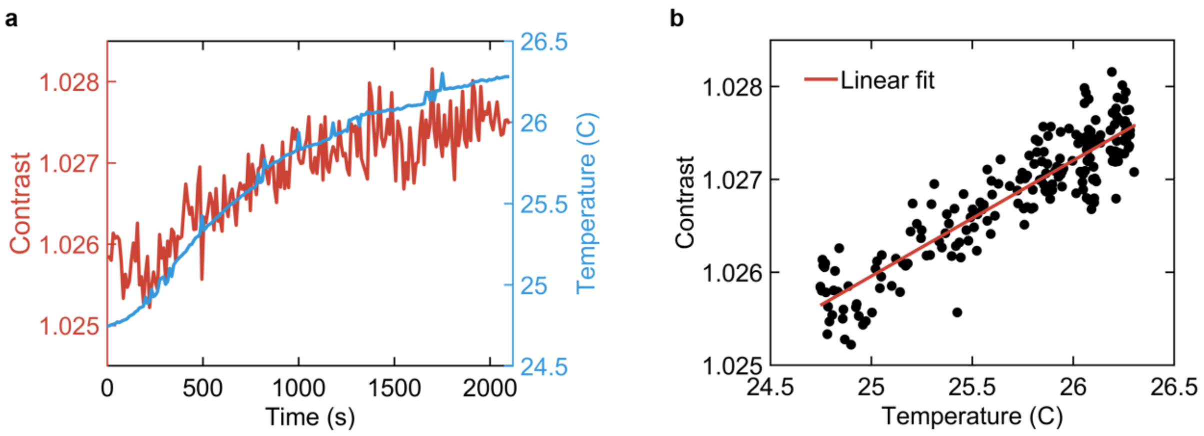

FIGURE S3. **a** The change in spin relaxation contrast (red curve) over time for increasing ambient temperature (blue curve) within ~1.5 °C. **b** Scatter plot of the spin relaxation contrast (C) versus the temperature (T) showing data (black points) and the liner fit (red line). Linear fit yields $\frac{\Delta C}{\Delta T} = 0.0012$ °C^{-1}

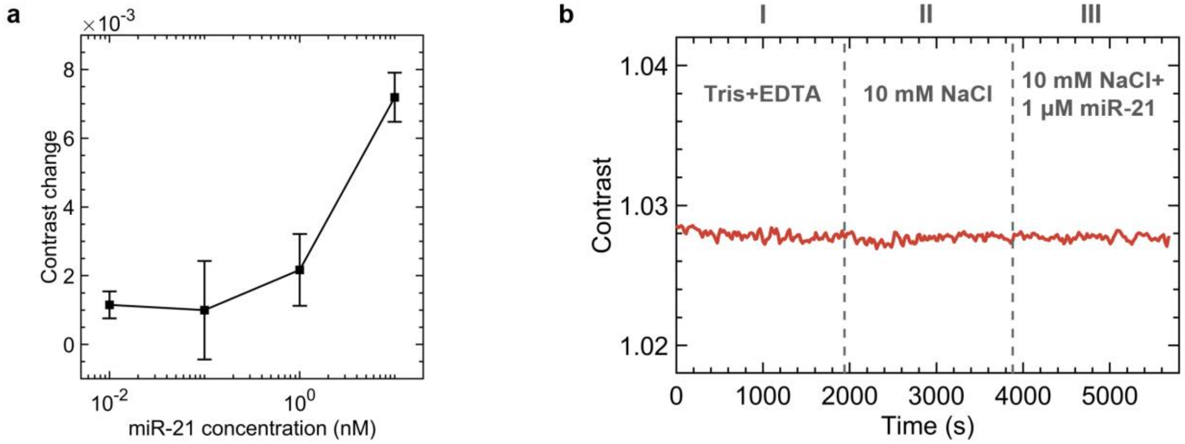

FIGURE S4. **a** A calibration curve showing the change in spin relaxation contrast as a function of miR-21 concentration. The contrast change is estimated by subtracting the signal average for solution I in Fig. 1d from the average of the last 5 measured points for solution II in Fig. 1d. **b** The change in spin relaxation contrast for sequential injection of: I) Tris-EDTA buffer solution, II) 10 mM NaCl, and III) 1μM miR-21 in 10 mM NaCl. Vertical dashed lines represent the time of the next solution injection.

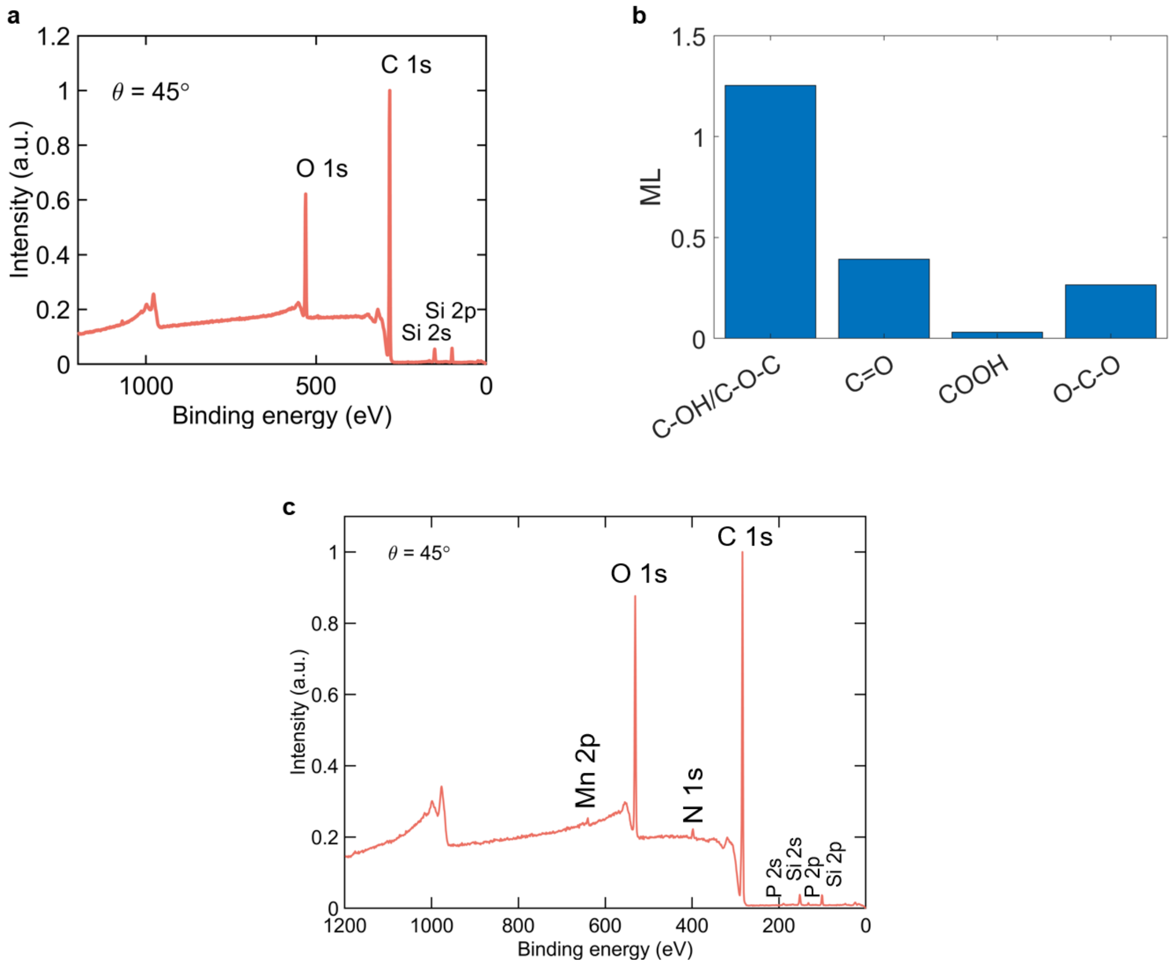

FIGURE S5. **a** X-ray photoelectron spectroscopy (XPS) survey spectrum of oxygenated diamond surface, measured at a photoelectron detection angle (θ) of 45°, showing O 1s and C 1s peaks. **b** Estimated coverages (monolayer (ML)) of oxygen groups on the diamond surface. The peak areas from the fitting- results in Fig. 2a are converted to coverages using conversion equations provided in Ref. [1]. The total coverage of oxygen groups is more than 1 ML indicating the full coverage of the surface with oxygen groups and crudeness of the evaluation method. **c** XPS survey spectrum of diamond surface after miR-21 treatment, showing O 1s, C 1s, Mn 2p, N 1s, P 2s and P 2p peaks. The observed Si 2s and Si 2p peaks in **a** and **c** are likely to be bulk contamination stemming from the synthesis of the diamond substrate.

Supplementary Note 1. Choice of additional counterions

We performed preliminary MD simulations of microRNA-21 (miR-21) with 48 replicas (100 ns) in the 298-348.5 K range using cubic box of 10.3 nm^3 that contains 35743 water molecules, 10 Mn, 13 Na and 12 Cl ions. From these simulations we get an excess ion number in a 3 nm shell around the nucleic acid of about 6.6 Mn, 3.7 Na, and -0.8 Cl (averages over 48 replicas). Note that the RNA charge is not fully neutralized at a 3 nm distance as the excess charge amounts to ≈18*e* vs the -21*e* of miR-21. In order not to overestimate the total number of Mn, we chose these numbers for the excess ions for neutralizing the miR-21: +6 Mn, +7 Na, -2 Cl.

For the simulated volume of the diamond system (≈2000 nm^3), in the absence of charges, the number of ions corresponding to [$MnCl_2$] = 5 mM and [NaCl] = 10 mM is 6 Mn, 12 Na and 24 Cl ions. In the case of neutral surfaces, the addition of miR-21 counterions leads to 12 Mn, 19 Na and 22 Cl ions. Furthermore, we estimate the excess number of ions due to the charged surfaces by solving the Poisson Boltzmann equation for a 1:1–2:1 electrolyte in the presence of a uniformly charged plane. In the case of -65*e* charged surface these numbers are: +28 Mn, +6 Na, -3 Cl. For the -32*e* charged surface they are: +12.5 Mn, +4 Na, -3 Cl.

The 6 simulated systems differ for the characteristics reported in Table S1, but all have the same box dimension of 10.097×10.097×(20.0+20.0) nm^3 (the additional 20 nm along *z*-axis is the empty buffer, see Fig 4a) and contain 65172 water molecules. 48 replicas were simulated with temperatures (K): 298.0, 298.8, 299.6, 300.4, 301.2, 302.0, 302.8, 303.6, 304.4, 305.2, 306.1, 306.9, 307.7, 308.5, 309.3, 310.1, 311.0, 311.8, 312.6, 313.5, 314.3, 315.1, 316.0, 316.8, 317.6, 318.5, 319.3, 320.1, 321.0, 322.0, 322.7, 323.5, 324.4, 325.2, 326.1, 327.0, 327.8, 328.7, 329.5, 330.4, 331.2, 332.1, 333.0, 333.8, 334.7, 335.6, 336.5, 337.3. The replica-exchange molecular dynamics (REMD) simulation time is 200 ns after which 1 ns normal MD simulations (i.e., without replica exchange) were performed.

TABLE S1. Surface composition and number of ions.

Groups on the diamond surface (single slab)	Number of protonated carboxyl groups	Charge (*e*) of single slab	Mn/Na/Cl (total)
345 Epoxy (C-O-C △)<br>435 Ether (O-C-O)<br>200 Carbonyl (C=O)<br>65 Carboxyl/-late ($COOH/COO^-$)			
	0	-65	68, 31, 16
	33	-32	37, 27, 16
	65	0	12, 19, 22
690 Hydroxyl (C-OH)<br>435 Ether (O-C-O)<br>200 Carbonyl (C=O)<br>65 Carboxyl/-late ($COOH/COO^-$)			
	0	-65	68, 31, 16
	33	-32	37, 27, 16
	65	0	12, 19, 22

Supplementary Note 2. Force field parameters for the diamond surface

The determination of atomic partial charges in chemical systems is of paramount importance for parameterizing force fields in classical molecular dynamics simulations, as these charges are known to have a sensitive impact [2, 3]. In this context, restrained electrostatic potential (RESP) derived charges for the surface atoms of diamond were calculated employing the CP2K software package [4]. All calculations were conducted within the framework of Density Functional Theory (DFT), employing the hybrid Gaussian and plane waves (GPW) approach [5] and applying periodic boundary conditions (PBC).

The Perdew–Burke–Ernzerhof exchange and correlation functional parameterized for solids (PBEsol) [6, 7] was used. The basis sets utilized were the optimized short-range double-ζ plus polarization [8] (DZVP-MOLOPT-SR), coupled with Goedecker-Teter-Hutter (GTH) pseudopotentials [9] optimized with PBE for all atoms. The energy cutoff for the auxiliary plane-wave basis was set to 800 Ry. Geometry and cell optimizations were carried out using Broyden–Fletcher–Goldfarb–Shanno (BFGS) minimization algorithm as implemented in CP2K. Convergence criteria were 10^{-4} Bohr for displacements and 10^{-4} Hartree/Bohr for the forces, respectively.

The system consisted of an *xy*-square diamond slab (see Fig. S6) with a thickness of 1 nm. The simulation box had dimensions of $2.02\times2.02\times3.0$ nm^3. Carbon atoms on one surface were saturated with hydrogen, while atoms on the opposite surface were functionalized with epoxy, ether, carbonyl, and carboxyl groups. A second system was generated by replacing epoxy groups with hydroxyl groups. RESP-derived charges for all atoms were evaluated after minimization of the systems using the PROPERTIES/RESP module implemented in CP2K [10]. Charge calculations were performed by restraining the charge value of bulk carbon atoms (dark grey in Fig. S6) to zero and constraining the charges of atoms to be equal per kind of atom: carbon atoms at the surface (violet in Fig. S6), epoxy oxygens, ether oxygens, etc. To maintain the system neutral, carboxyl groups were protonated. However, the final charges of the carboxyl groups were determined by adopting the charges of the deprotonated carboxyl groups of the acidic amino acids present in the used force field (Table S2).

The DFT-minimized cells were replicated 25 times (5×5) to obtain the slab(s) used in the MD simulations. Some groups were substituted by hand to better replicate the estimates from the XPS measurements.

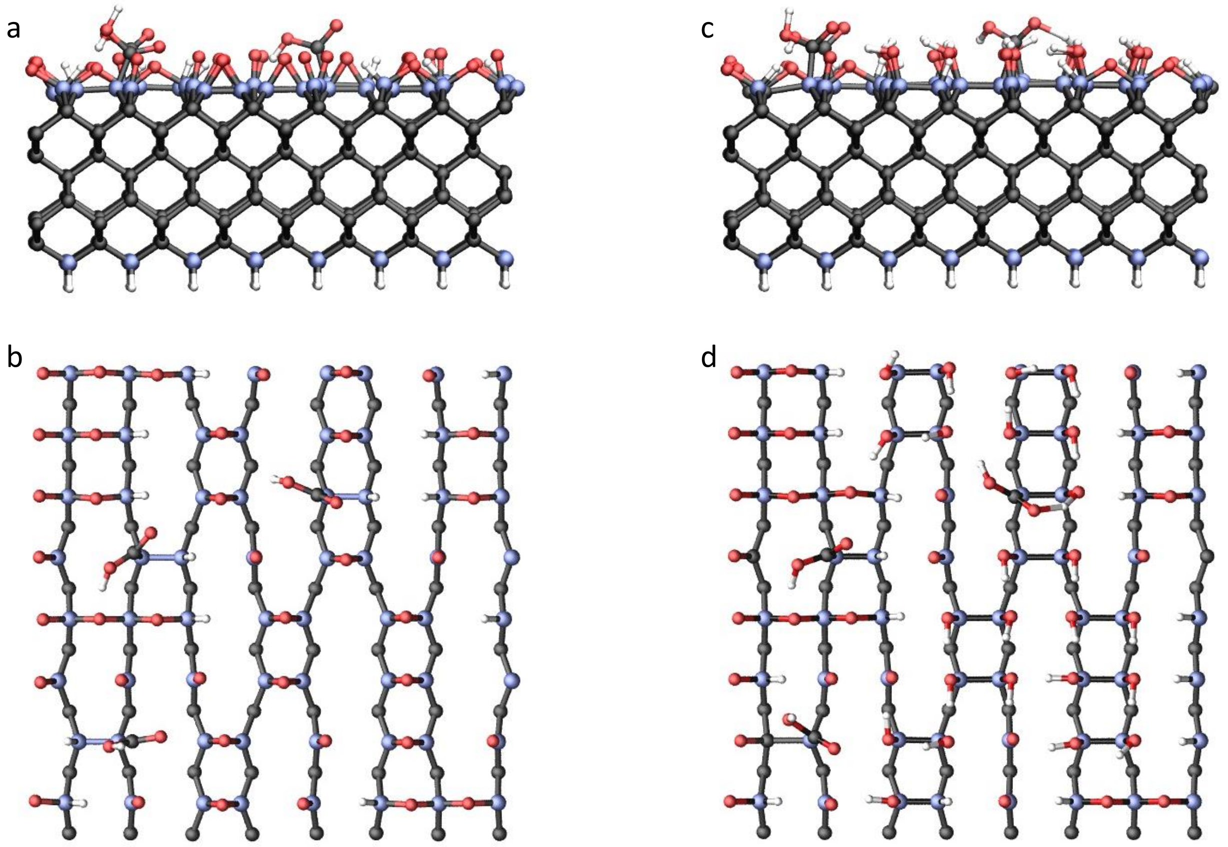

FIGURE S6. Panels **a** and **b**: side and top view of the DFT-minimized diamond slab with epoxy groups. In the top view only the first layer of carbon atoms is shown together with the functional groups. Panels **c** and **d**: same as **a** and **b** but for the diamond slab with epoxy groups replaced by hydroxyl groups.

TABLE S2. Force field parameters of the diamond model

Group name†	Atom name	Atom type‡	Charge	Frozen	Notes
DCY					Carboxylate group<br>Bond, angle and dihedral parameters taken from GLU/ASP residue
	CUD	CUD	0.0000	Y	
	CCD	CTD	-0.1600	N	
	CED	C	0.8000	N	0.8054/0.7994 in Amber ff99
	OFD	O2	-0.8200	N	-0.8188/-0.8014 in Amber ff99 for Glu and Asp respectively
DCW					Carboxyl group<br>Bond, angle and dihedral parameters taken from protonated GLU
	CUD	CUD	0.0000	Y	
	CCD	CTD	0.0970	N	
	CED	C	0.6801	N	As in Amber ff99 for protonated Glu
	OFD	O	-0.5838	N	As in Amber ff99 for protonated Glu
	OFD	OH	-0.6511	N	As in Amber ff99 for protonated Glu
	HFD	HO	0.4641	N	As in Amber ff99 for protonated Glu
DOH					Hydroxyl group(s)<br>Bond, angle and dihedral parameters taken from SER
	CUD	CUD	0.0000	Y	
	CCD	CTD	0.2200	Y	
	OHD	OH	-0.6400	N	-0.6761 in Amber ff99 for Ser
	HOD	HO	0.4200	N	0.4275 in Amber ff99 for Glu
DCO					Carbonyl group<br>Bonds and angle parameters taken from peptide carbonyl group
	CUD	CUD	0.0000	Y	
	CKD	Cd	0.3500	Y	
	OKD	O	-0.3500	N	
DPX					Epoxy group
	CPD	CPD	0.1400	Y	
	OPD	OPD	-0.2800	Y	
DOE					Ether group(s)
	OED	OEDl	-0.3700	Y	
	OED	OEDc	-0.4600	Y	
	CED	CED	0.5000	Y	
	CHD	CHDe	-0.0500	Y	
	HCD	HCDe	0.1500	Y	
DCH					-CH<br>This is always next to a DCY or DCW to saturate one of the 3 carbons
	CHD	CHD	-0.1500	Y	
	HCD	HCD	0.1500	Y	
DCB					Topmost layer of bulk carbon atoms
	CBD	CBD	0.0000	Y	
DCI					Bulk carbon atoms
	CID	CID	0.0000	Y	

† See Fig. S7 for group names and atom names

‡ Lennard-Jones parameters assigned as in the AMBER ff14SB-bsc1 force field. All non-bonded interactions are excluded among diamond carbon atoms.

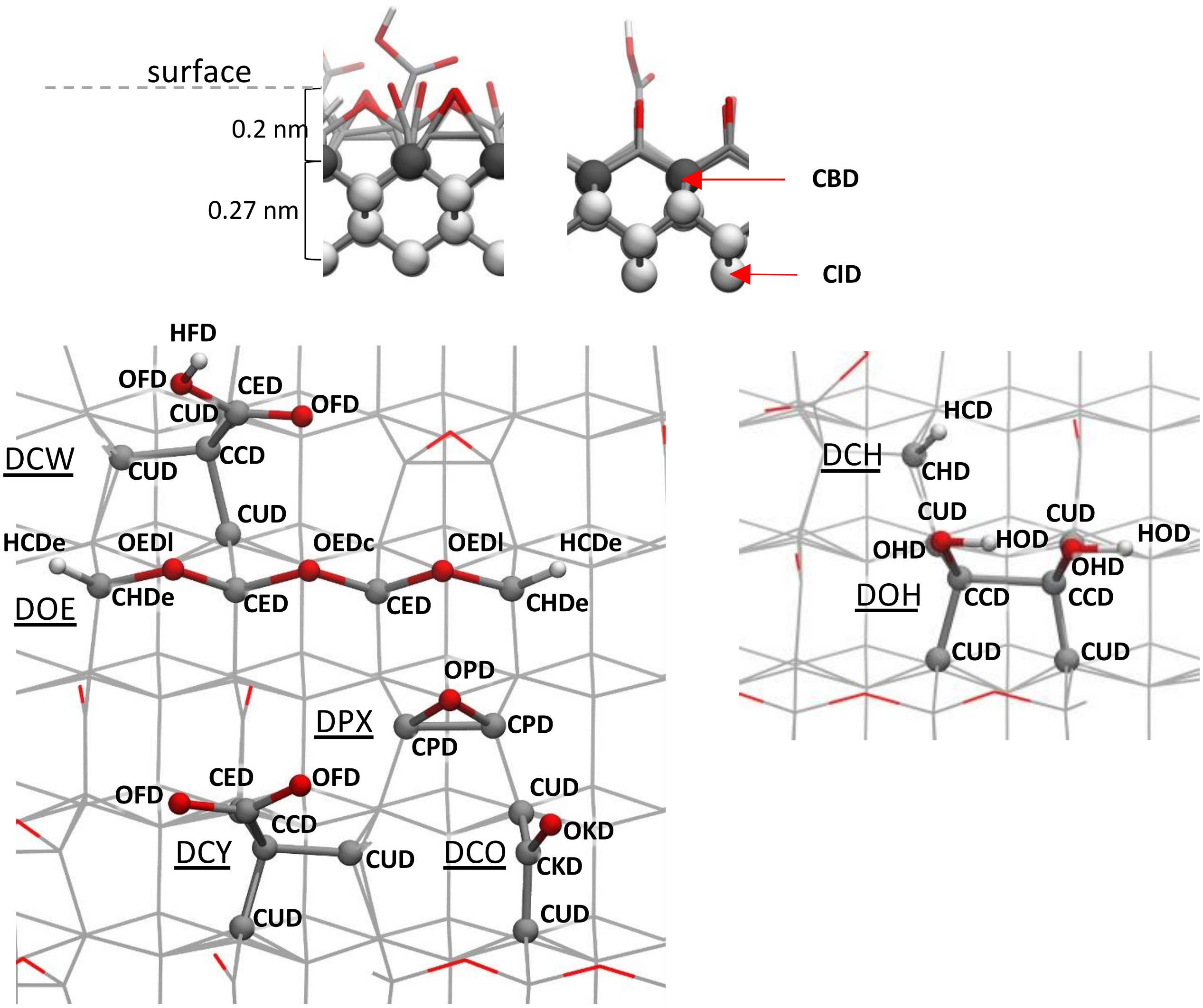

FIGURE S7. Groups and atom names of the diamond models.

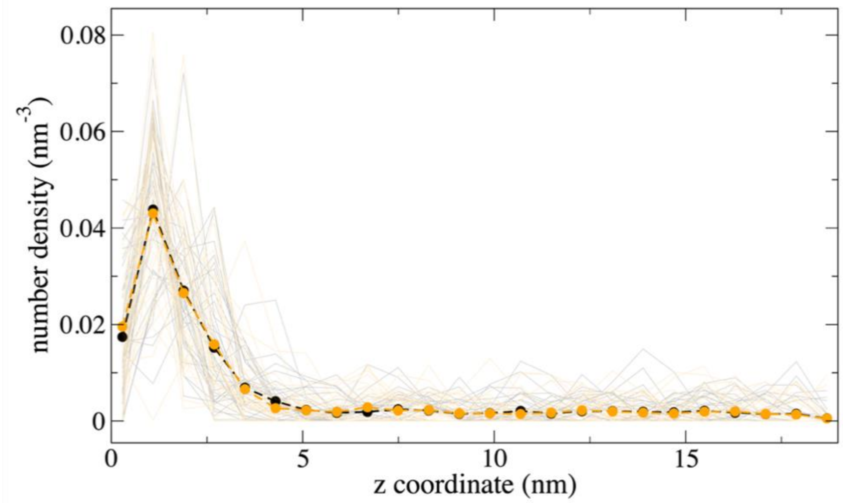

FIGURE S8. Number density profiles of Mn ions in the simulated temperature range (298-337 K). Temperature does not play a relevant role in the ion distribution after averaging. The graph shows, in the case of the 0*e* hydroxyl system, the comparison between the Mn^{2+} densities at different temperatures (black) and those after cooling all replicas to 298 K by additional 1 ns equilibration (orange). The circles are the average values while the profiles for each of the replicas are shown in light colors.

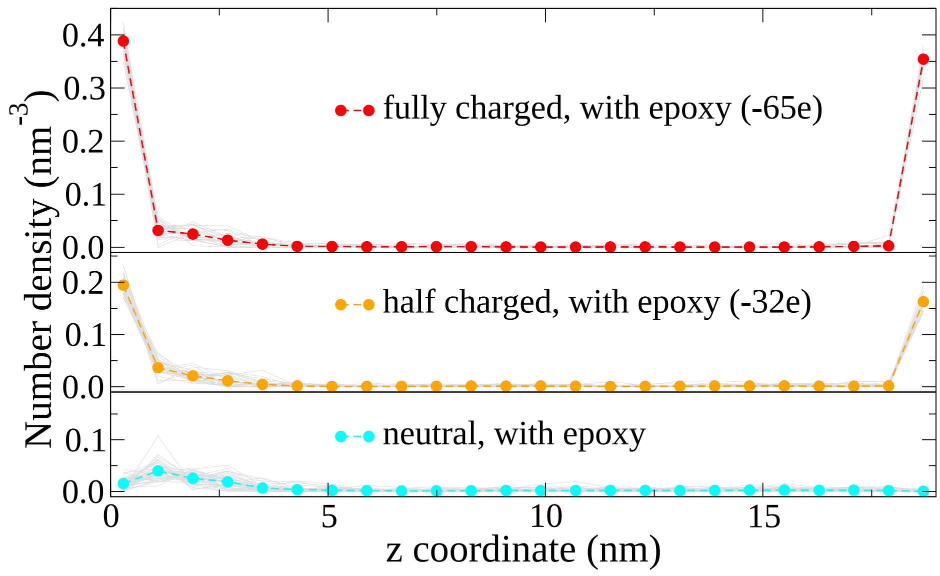

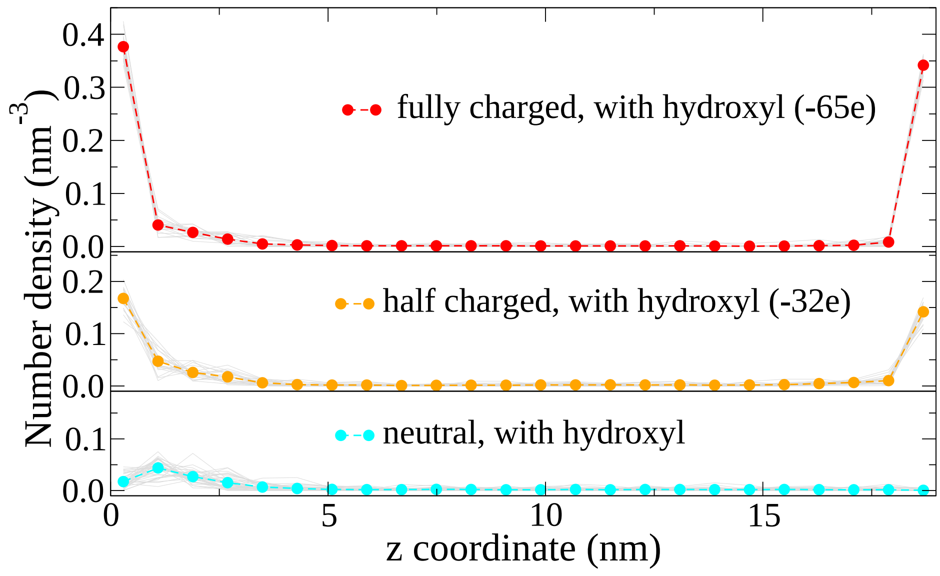

FIGURE S9. Top: Number density profiles of Mn ions along the *z*-axis when miR-21 is adsorbed onto the surface containing epoxy groups. Solid circles show the average over the replicas, while the grey lines are the profiles for each replica. Bottom: same as top but for the systems with epoxy groups replaced by hydroxyl groups.

Supplementary Note 3. Statistics of miR-21 and diamond surface contact events

The contact event of miR-21 to the diamond surface occurs faster than for the neutral surfaces (see Table S3), leading to 80% of replicas with adsorption. In the charged cases there is not much difference between the fully charged (-65*e*) and the half charged (-32*e*) case. The charged surfaces with epoxy seem to be more easily contacted (more than 50%, up to 58%) than the ones with hydroxyl (at 44%), though the statistics is limited to be able to strongly differentiate between the two cases.

Fig. S10 shows the involvement of each component of the miR-21 and the diamond surface in the adsorption process. The left side graphs report how much each nucleotide is interacting with the surface. The analysis highlights a relevant role of the 3' terminal nucleotide in the contact with the surface. Being at the terminal side, this nucleotide is less engaged in interactions with the rest of the miR-21 and thereby is freer to reach the functional groups on the surface. By contrast, the nucleotide at the 5' does not show a similar behaviour, in part, as verified by visual inspection, because it is in general in an internal position of the structure. The same percentages are calculated in the middle graphs for each base type and for all the phosphate groups. The guanine bases seem to be in some cases less involved in interactions with the surface. Finally, the distribution of diamond surface components interacting with the miR-21 generally follows the overall surface composition (grey bars). The direct (<0.4nm) interaction is more frequent with neutral than with anionic carboxyl groups. The epoxy groups are slightly under-represented in the interacting pool, while the hydroxyl is over-represented. The ether groups are generally favoured apart from the fully charged case with hydroxyl groups. Overall, these variations are not large, implying that there is no strong preference of a particular functional group for interaction with the RNA.

A general comment to this analysis should be added. Thanks to the REMD approach, we account for several configurations of the miR-21, to avoid possible biases due to the choice of a particular configuration. Despite its significant biological role, only limited research is available on the structural aspects of mature miR-21. Ref. [11] concludes that miR-21 can adopt hairpin and homoduplex structures depending on the concentration. To derive meaningful quantitative insights on the propensity of each RNA component to interact with the surface, our MD study needs to be expanded to: i) attain exhaustive exploration of the configurational landscape; ii) include several copies of the RNA strand, in order to study their interactions among them and with the surface.

TABLE S3. Statistics of contact events between miR-21 and diamond surface

System (charge)	# repl with ads† @100ns	# repl with ads (%)† @200ns	# oxygen in contact with miR-21‡	# miR-21 atoms in contact with surface §
Epoxy (-65e)	7	25 (52%)	20.0	66.3
Epoxy (-32e)	9	28 (58%)	32.3	101.0
Epoxy (0)	20	39 (81%)	29.5	88.4
Hydroxyl (-65e)	11	21 (44%)	23.9	61.6
Hydroxyl (-32e)	11	21 (44%)	37.6	88.8
Hydroxyl (0)	19	38 (79%)	44.1	104.0

† Number of replicas that feature miR-21 adsorption.

‡ Average number of surface oxygen atoms that are within 0.4 nm from the miR-21 (non-hydrogen atom only) in the simulations with adsorption. The total number of oxygen atoms is higher in the hydroxyl model (1455 vs 1110 in the case of epoxy models), partially explaining the larger number of contacts for these models.

§ Average number of miR-21 non-hydrogen atoms closer than 0.5 nm to the diamond surface in the simulations with adsorption.

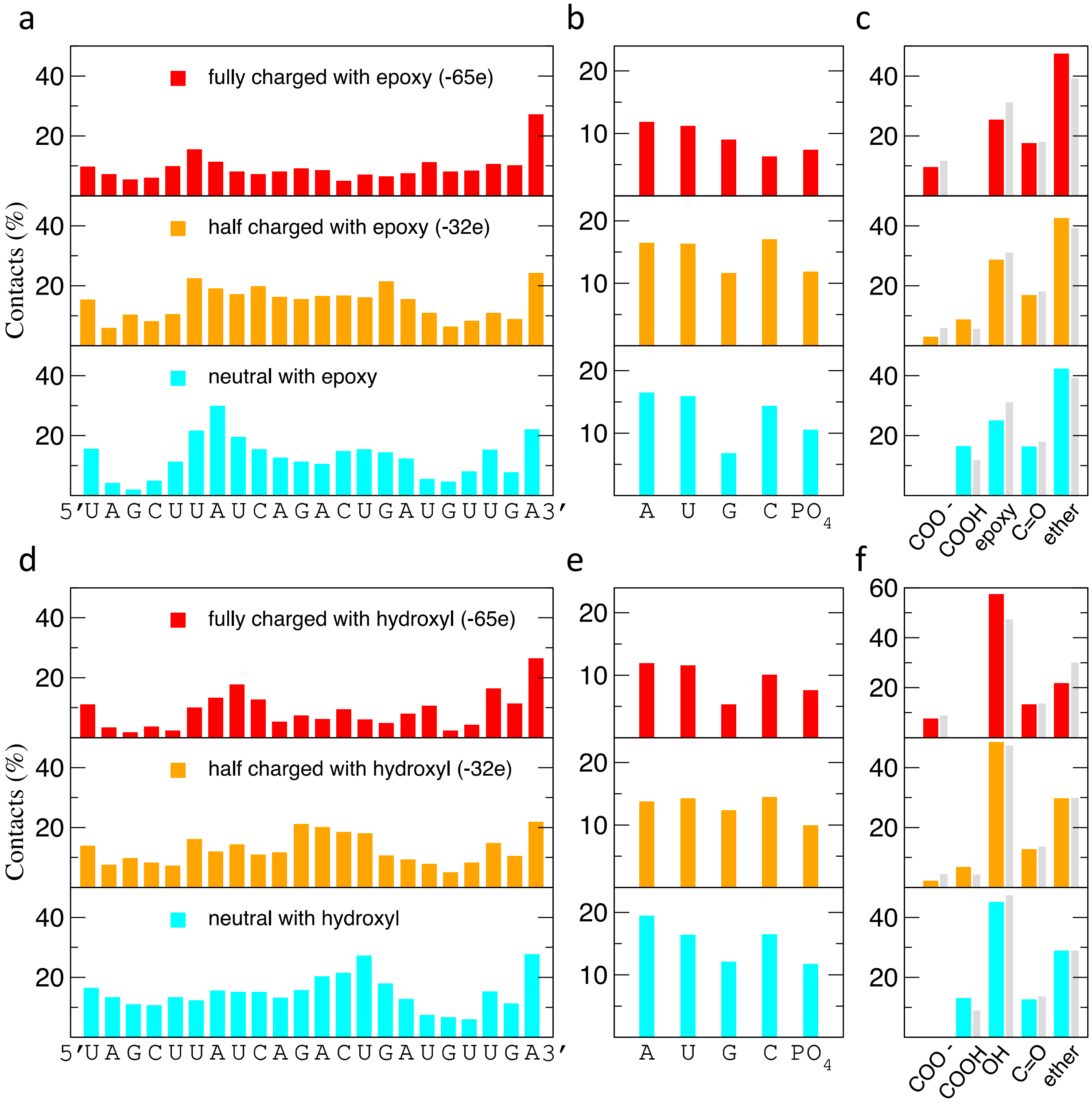

FIGURE S10. **a** Average percentage of atoms of each miR-21 nucleotide involved in the interaction with the diamond surface with epoxy groups. Reported values are calculated as the number of (non-hydrogen) atoms of each nucleotide closer than 0.5 nm to the diamond surface divided by the number of atoms in the nucleotide. The analysis is done for simulations with miR-21 adsorption events, and the percentage shown are averages over the simulation time and the 48 replicas. **b** The same contact events as in the left panels but summarized according to each base type and phosphate groups (PO_4). **c** Overall contribution of each surface functional group to the interaction with miR-21. The coloured bars are the percentage of oxygen atoms of a particular group over all oxygen atoms involved in the interaction with miR-21, where an oxygen atom is considered in contact if the distance from miR-21 is less than 0.35 nm. The grey bars show how each group contributes to the overall surface composition and is calculated as the percentage of oxygen atoms of each group over all surface oxygen atoms. **d, e, f** Same as **a, b** and **c** but for the systems featuring the surface with hydroxyl groups replacing epoxy groups.

Supplementary Note 4. Estimation of $\langle \boldsymbol{B}_{\perp}^{2} \rangle$

$\langle B_{\perp}^{2} \rangle$ is determined by the distribution of paramagnetic ions in a solution. We consider a magnetic dipole and a single NV center as shown in Fig. S11. The magnetic field produced by a magnetic dipole $\vec{m}$ is

$$\vec{B}(\vec{r}) = \frac{\mu_0}{4\pi}\left(\frac{3\,\vec{r}(\,\vec{m}\cdot\vec{r})}{r^5} - \frac{\vec{m}}{r^3}\right). \tag{S1}$$

μ_0 is the vacuum permeability constant and $\vec{r}$ is the distance vector from the position of the magnetic dipole to where the magnetic field is evaluated. Considering a paramagnetic ion, $\vec{m}$ is given as:

$$\vec{m} = \frac{g_e\mu_B}{\hbar}\vec{\boldsymbol{S}} = \gamma_e\,\vec{\boldsymbol{S}},$$

where g_e is the gyromagnetic ratio, μ_B is the Bohr magneton, γ_e is the electron gyromagnetic factor and $\vec{\boldsymbol{S}}$ is the spin vector operator.

For a given configuration of paramagnetic ions, the power spectral density of the magnetic noise is obtained by averaging $B_{\perp}^{2}$ over the paramagnetic spin states. This leads to the following relations, where $k = x, y, z$, $\hat{r} = \vec{r}/r$, and $\langle \ldots \rangle_S$ implies taking the trace of the spin operators over a mixed-state density matrix:

$$\langle B_k(\vec{r})\cdot B_k(\vec{r})\rangle_S = \left(\frac{\mu_0}{4\pi}\gamma_e\right)^2 \frac{1}{r^6}\langle \boldsymbol{S}_k^2 + 9\,\hat{r}_k^2\big(\vec{\boldsymbol{S}}\cdot\hat{r}\big)^2 - 6\,\hat{r}_k\boldsymbol{S}_k\big(\vec{\boldsymbol{S}}\cdot\hat{r}\big)\rangle_S,$$

$$\langle \boldsymbol{S}_k^2\rangle_S = \frac{s(s+1)}{3}\hbar^2,$$

$$\langle\big(\vec{\boldsymbol{S}}\cdot\hat{r}\big)^2\rangle_s = \frac{s(s+1)}{3}\hbar^2,$$

$$\langle\,\hat{r}_k\boldsymbol{S}_k\big(\vec{\boldsymbol{S}}\cdot\hat{r}\big)\rangle_s = \frac{s(s+1)}{3}\hbar^2\hat{r}_k{}^2,$$

where the quantum spin number s is $5/2$ for Mn^{2+}. Collecting all terms and summing over the positions r_I of the Mn ions we get:

$$\langle B_k^2(\vec{r})\rangle = C_s\,\langle \textstyle\sum_{I\epsilon \mathrm{Mn}} \frac{1+3\hat{k}_I^2}{r_I^6}\rangle,$$

were $\hat{k}_I$ is $\hat{r}_k$ of the Ith ion and

$$C_s = \frac{s(s+1)}{3}\left(\frac{\mu_0}{4\pi}\gamma_e\hbar\right)^2 = 1.00574\times10^{-5}\ \mathrm{nm^6T^2}.$$

The four possible orientations of NV in the {100} diamond surface (see Fig. S11) are:

$$\hat{u}^{1,2} = \frac{1}{\sqrt{3}}\big(\pm\sqrt{2}, 0, -1\big), \qquad \hat{u}^{3,4} = \frac{1}{\sqrt{3}}\big(0, \pm\sqrt{2}, 1\big).$$

The orthogonal components of a generic vector $\vec{V}$ to these directions can be found as:

$$\vec{V}_{\perp}^{j} = \vec{V} - \vec{V}\cdot\hat{u}^j\,\hat{u}^j,$$

leading to

$$\big|\vec{V}_{\perp}^{1,2}\big|^2 = \frac{1}{3}V_x^2 + V_y^2 + \frac{2}{3}V_z^2 \mp \frac{2\sqrt{2}}{9}V_xV_z\ , \qquad \big|\vec{V}_{\perp}^{3,4}\big|^2 = V_x^2 + \frac{1}{3}V_y^2 + \frac{2}{3}V_z^2 \mp \frac{2\sqrt{2}}{9}V_yV_z.$$

If $\langle B_{x,y}B_z\rangle_S = 0$, then

$$\left|\vec{B}_{\perp}^{1,2}\right|^2 = \frac{1}{3}B_x^2 + B_y^2 + \frac{2}{3}B_z^2,$$
$$\left|\vec{B}_{\perp}^{3,4}\right|^2 = B_x^2 + \frac{1}{3}B_y^2 + \frac{2}{3}B_z^2.$$

Assuming a uniform distribution of the 4 possible NV orientations we can write

$$\langle B_{\perp}^2\rangle = \frac{2}{3}\left(\langle B_x^2\rangle + \langle B_y^2\rangle + \langle B_z^2\rangle\right)$$

so that

$$\langle B_{\perp}^2(\vec{r})\rangle = 4C_s\,\langle \textstyle\sum_{I\epsilon \mathrm{Mn}} \frac{1}{r_I^6}\rangle.$$

For each MD snapshot we calculated the $\langle B_{\perp}^2(\vec{r})\rangle$ and averaged it over the simulation time. Alternatively, $\vec{B}$ can be estimated from Eq. (S1) assigning a random orientation to each ion at each MD snapshots, and then taking the square of the NV-orthogonal component. We verified that the two approaches lead to the same results within numerical accuracy.

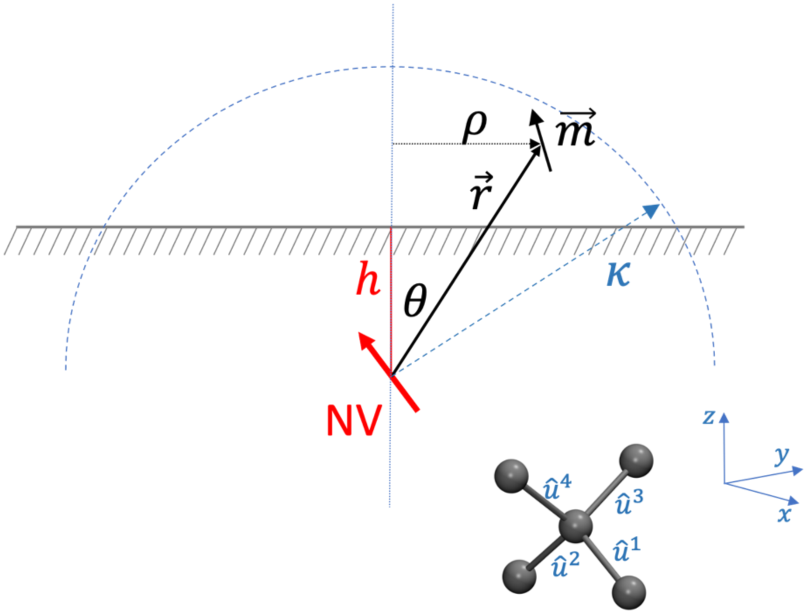

FIGURE S11. Geometry of the magnetic dipole and the NV for the estimation of $\langle B_{\perp}^2\rangle$. In the bottom of the picture the four possible orientations of a NV are shown.

We accounted for the periodic boundary conditions by including in the sum also the images of the ions up to a certain cutoff κ (see Fig. S11). We can correct for $\kappa \to \infty$ by adding the contribution of a uniform bulk (n) and surface density (σ) of Mn^{2+} for $|\vec{r}| > \kappa$. To find this correction we observe that for a distribution of Mn ions isotropic in x and y we have up to a certain cutoff κ

$$\langle B_{\perp}^2\rangle = 4C_s \int \frac{n(\vec{r})}{r^6}\,dV \qquad \text{(S2)}$$
$$= 4\,C_s \int_h^{\kappa} dz\, n(z) \int_0^{\sqrt{\kappa^2 - z^2}} 2\pi\rho d\rho \frac{1}{(z^2+\rho^2)^3} = 2\pi\, C_s \int_h^{\kappa} dz\, n(z) \left(\frac{1}{z^4} - \frac{1}{\kappa^4}\right).$$

Substituting $n(z) = n + \sigma\,\delta(z-h)$ yields

$$\langle B_\perp^2 \rangle = 2\pi\, C_s \int_h^\kappa dz\,(n + \sigma\delta(z-h))\left(\frac{1}{z^4} - \frac{1}{\kappa^4}\right) = 2\pi C_S\left(\frac{n}{3h^3} - \frac{4}{3}\frac{n}{\kappa^3} + \frac{nh}{\kappa^4} + \frac{\sigma}{h^4} - \frac{\sigma}{\kappa^4}\right).$$

The correction ($corr$) is then

$$corr = 2\pi C_S\left(\frac{4}{3}\frac{n}{\kappa^3} - \frac{nh}{\kappa^4} + \frac{\sigma}{\kappa^4}\right).$$

We used $\kappa = 30$ nm, $n = 3.011 \times 10^{-3}\ \mathrm{nm}^{-3}$ and $\sigma = .6376\ \mathrm{nm}^{-2}, .3139\ \mathrm{nm}^{-2}$ and 0 for the totally charged (65e), half charged (32e) and neutral surface respectively, n corresponding to the bulk concentration (0.5 mM) and σ to the charge of the surface divided by the surface area of the diamond slab ($10.097 \times 10.097\mathrm{nm}^2$), with the approximation that the Mn ions will completely counteract the surface charge. The values for this correction are $5.7 \times 10^{-11}\mathrm{T}^2,\ 3.2 \times 10^{-11}\mathrm{T}^2, .8 \times 10^{-11}\mathrm{T}^2$ for the three systems respectively.

For completeness, we also report the expressions for the separate components, in the case of a xy isotropic ion distribution:

$$\langle B_x^2 \rangle + \langle B_y^2 \rangle = C_S \int \frac{n(\vec{r})}{r^6}(2 + 3\sin^2\theta)\, dV = \pi C_S \int_h^\kappa dz\, n(z)\left(\frac{3}{2z^4} + \frac{z^2}{\kappa^6} - \frac{5}{2\kappa^4}\right),$$

$$\langle B_z^2 \rangle = C_S \int \frac{n(\vec{r})}{r^6}(1 + 3\cos^2\theta)\, dV = \pi C_S \int_h^\kappa dz\, n(z)\left(\frac{3}{2z^4} - \frac{z^2}{\kappa^6} - \frac{1}{2\kappa^4}\right).$$

The values of $\langle B_\perp^2 \rangle$ from the MD simulations refer to a system with a single miR-21 on a $10.097 \times 10.097\ \mathrm{nm}^2$ diamond slab, the NV position being aligned with the center of mass of the microRNA molecule. We can extrapolate to the case of complete coverage with the following argument. Each miR-21 recruits 8 Mn^{2+} in a ~4-nm thick layer and covers on average ~10 nm^2. The extra Mn^{2+} number density Δn in this 4-nm layer is thereby 0.2 nm^{-3}, which upon integration of Eq. (S2), yields $\langle B_\perp^2 \rangle \sim .9 \times 10^{-8}\ \mathrm{T}^2$.

Supplementary References

[1] Kono, S. et al. Carbon 1s X-ray photoelectron spectra of realistic samples of hydrogen-terminated and oxygen-terminated CVD diamond (111) and (001). *Diamond and Related Materials* **93** 105–130 (2019).
[2] Dupradeau, F.-Y. et al. The R.E.D. tools: advances in RESP and ESP charge derivation and force field library building. *Physical Chemistry Chemical Physics* **12** 7821 (2010).
[3] Golze, D., Hutter, J. & Iannuzzi, M. Wetting of water on hexagonal boron nitride@Rh(111): a QM/MM model based on atomic charges derived for nano-structured substrates. *Physical Chemistry Chemical Physics* **17** 14307–14316 (2015).
[4] Kühne, T. D. et al. CP2K: An electronic structure and molecular dynamics software package - Quickstep: Efficient and accurate electronic structure calculations. *The Journal of Chemical Physics* **152** (2020).
[5] VandeVondele, J. et al. Quickstep: Fast and accurate density functional calculations using a mixed Gaussian and plane waves approach. *Computer Physics Communications* **167** 103–128 (2005).
[6] Perdew, J. P. et al. Restoring the Density-Gradient Expansion for Exchange in Solids and Surfaces. *Physical Review Letters* **100** (2008).
[7] De La Pierre, M. et al. Performance of six functionals (LDA, PBE, PBESOL, B3LYP, PBE0, and WC1LYP) in the simulation of vibrational and dielectric properties of crystalline compounds. The case of forsterite Mg_2SiO_4. *Journal of Computational Chemistry* **32** 1775–1784 (2011).
[8] VandeVondele, J. & Hutter, J. Gaussian basis sets for accurate calculations on molecular systems in gas and condensed phases. *The Journal of Chemical Physics* **127** (2007).
[9] Krack, M. Pseudopotentials for H to Kr optimized for gradient-corrected exchange-correlation functionals. *Theoretical Chemistry Accounts* **114** 145–152 (2005).
[10] Golze, D., Hutter, J. & Iannuzzi, M. Wetting of water on hexagonal boron nitride@Rh(111): a QM/MM model based on atomic charges derived for nano-structured substrates. *Physical Chemistry Chemical Physics* **17** 14307–14316 (2015).
[11] Belter, A. et al. Mature MiRNAs Form Secondary Structure, which Suggests Their Function beyond RISC. *PLoS ONE* **9** e113848 (2014).
